# Research progress of immunotherapy against anaplastic thyroid cancer

**DOI:** 10.3389/fonc.2024.1365055

**Published:** 2024-03-26

**Authors:** Jiaqian Chen, Zuixuan Xiao, Hongyan Wu

**Affiliations:** Department of Endocrinology, The First Affiliated Hospital of Yangtze University, Jingzhou, China

**Keywords:** anaplastic thyroid cancer, immunotherapy, tumor microenvironment, immune checkpoint inhibitors, cellular immunotherapy

## Abstract

Anaplastic thyroid cancer (ATC) is the most aggressive type of thyroid cancer. While ATC is rare, its mortality is high. Standard treatments, such as surgery, radiotherapy, and chemotherapy, have demonstrated limited efficacy in managing ATC. However, the advent of immunotherapy has significantly improved the prognosis for patients with ATC. Immunotherapy effectively targets and eliminates tumor cells by using the power of the body’s immune cells. The neoantigen is an atypical protein generated by somatic mutation, is exclusively observed in neoplastic cells, and is devoid of central tolerance. Neoantigens exhibit enhanced specificity towards tumor cells and display robust immunogenic properties. Currently, neoantigen therapy is primarily applied in immune checkpoint inhibitors and cellular immunotherapy, encompassing adoptive immunotherapy and tumor vaccines. This study discusses the mechanism, tumor microenvironment, clinical trials, adverse events, limitations and future directions associated with ATC immunotherapy.

## 1 Introduction

Also known as undifferentiated thyroid carcinoma, anaplastic thyroid carcinoma (ATC) is the most malignant thyroid cancer and one of the most aggressive solid tumors (1). ATC accounts for <2% of thyroid cancers, but >40% of patients have large primary tumors, extrathyroidal spread, and local and distant metastases at the time of diagnosis (2). The median survival after initial diagnosis is approximately four months, and one in five patients survives >12 months. Furthermore, the 5-year survival rate is close to 0% (3). The standard ATC treatment includes surgery, radiotherapy, and chemotherapy. However, ATC is extremely malignant, and the effect of treatment is limited due to its invasiveness, lack of differentiation, and chemoresistance (4, 5). While ATC features gene mutations the incidence rate is lower than that of differentiated thyroid cancer. The signaling pathways in ATC are mainly MAPK, PI3K–AKT–mTOR, and JAK–STAT, which contain important targets such as RET and EGFR. Targeted drugs such as sorafenib, lenvatinib, dabrafenib, and trametinib have exerted some effects, but drug resistance is the main issue that should be solved (6). Therefore, safer and more effective treatments are needed to improve the prognosis of patients with ATC. The use of immunotherapy to treat solid tumors, including ATC, has become a promising treatment option (7). Immunotherapy has developed rapidly in recent years and yielded significant results in reducing disease recurrence and prolonging patient survival. Moreover, various levels of combined therapy can synergistically combat tumors and minimize side effects. This article reviews the current immune mechanism and immunotherapy of ATC.

## 2 ATC immunotherapy mechanisms

### 2.1 Fundamentals of immunotherapy

Tumor immunotherapy uses the body’s immune cells to eliminate tumor cells, with T cells being the main effector cells. Tumor elimination by T cells begins with the recognition of tumor antigens (8), which are self-molecules altered by gene mutation, protein truncation, protein misfolding, or abnormal post-translational modification (9). Tumor antigens are divided into tumor-associated antigens (TAAs) and tumor-specific antigens (TSAs) (10).TAAs are macromolecules found on the surface of tumor cells and can also be found in normal tissues. Nevertheless, TAAs are highly expressed in tumors, most of which have central tolerance and weak immunogenicity (11). TSAs, or neoantigens, are not expressed in normal somatic cells and do not exhibit central tolerance. TSAs are more specific to tumor cells and have strong immunogenicity. Neoantigens can be presented on the cell surface and subsequently recognized by T cells under the action of major histocompatibility complex (MHC) molecules, causing T cell activation and promoting T cell-mediated attack and removal of tumor cells (12). Neoantigens contribute to tumor-specific immune responses and have been used as targets for novel, precise, and personalized tumor immunotherapy (13).Currently, neoantigen therapy is mainly used in immune checkpoint inhibitors (ICIs) and cellular immunotherapy (adoptive immunotherapy and tumor vaccines).

### 2.2 Relationship between tumor microenvironment and ATC

The tumor microenvironment (TME) refers to the environment required for tumor cell appearance and development and contains tumor cells, extracellular matrix, immune cells, and the cytokines, metabolites, and exosomes they release (14). Different organs have biologically unique microenvironments, and different thyroid cancer types also have greatly differing immunological TMEs (15). Immune cells in the ATC TME mainly consist of tumor-associated macrophages (TAMs), tumor-associated mast cells (MCs), dendritic cells (DCs), natural killer cells (NKs), cytotoxic T cells (CTLs), and regulatory T cells (Tregs) (16). However, some scientists believe that ATC only involves cancer cells, macrophages, and vascular endothelial cells and almost no other cells (17). Due to the low prevalence and low possibility of surgery for ATC, most of the current studies address the role of the phenotypic characteristics of tumor-associated immune cells in the pathogenesis or progression of papillary thyroid carcinoma, whereas there are few studies on ATC.

Thyroid cancer has an extremely wide range of TAM density, with TAM density being the highest in ATC (18). TAMs can directly promote tumor occurrence, development, and metastasis by releasing various inflammatory factors, growth factors, and matrix proteases. TAMs can also indirectly promote tumor progression by mediating tumor angiogenesis and immunosuppression (19, 20). TAMs are mainly divided into M0, M1, and M2 types (21). M0 TAMS are usually dormant, while the proinflammatory M1 TAMs mainly produce cytokines such as tumor necrosis factor-α (TNF-α) and interleukin-1 (IL-1), which inhibit and kill tumor cells. The anti-inflammatory M2 TAMs produce factors such as IL-10 and IL-13, which promote tumor formation and development (22). In ATC, TAMs account for >50% of nuclear cells, and the M2 type is predominant (17). M2-like TAMs accelerate ATC cell metastasis by activating the insulin receptor (IR)-A/insulin-like growth factor 1 receptor (IGF1R)-mediated PI3K–AKT–mTOR pathway and upregulating IGF-1/IGF-2 (23). It is also believed that only ATC contains a branched TAM network to promote ATC invasion through metabolic and nutritional functions (17).

The ATC microenvironment has significantly increased tumor-infiltrating lymphocytes (TILs) as compared with normal thyroid tissues, and most of them are CD8+T cells (24). However, only one investigator demonstrated that CD8+ T cells enhance their killing effect on ATC cells by secreting granzyme, TNF-α, and interferon-γ (IFN-γ) (25). In patients with ATC, NKs kill tumor cells by inducing apoptosis via the release of cytolytic granules (26). MCs are the first immune cells recruited during inflammation and are associated with ATC aggressiveness (27). ATC has a higher number of MCs than other forms of thyroid cancer. By producing IL-6, TNF-α, and CXC chemokine ligand 8 (CXCL8)/IL-8, MCs aid epithelial–mesenchymal transition in ATC cells (28). Galectin-9 aids MC support of ATC cell adhesion, angiogenesis, metastasis, and tumor immune escape (29). Most of the body’s professional antigen-presenting cells are termed DCs, yet the amount of DCs in ATC is much lower than that of other thyroid cancers (30, 31).

Soluble mediators such as cytokines and chemokines are mainly released by tumor-infiltrating immune cells and can also be released by cancer cells. ATC cells secrete CXCL9 and CXCL10, which are chemotactic to T cells (24). IL-4 and IL-10 upregulate the anti-apoptotic proteins B-cell lymphoma-2 (Bcl-2) and Bcl-xL to promote ATC cell progression and chemotherapy resistance (32). CXC chemokine receptor 4 (CXCR4) is involved in the local invasion and distant metastasis of ATC cells mediated by stromal cell-derived factor (33). The transcription factor CREB3L1 activates the extracellular matrix signaling pathway, maintains the fibroblast-like characteristics of ATC cells, reshapes the tumor interstitial microenvironment, and drives ATC malignant transformation (34). ATC is also linked to elevated levels of other cytokines, including IL-6, IL-1, granulocyte colony-stimulating factor, macrophage colony-stimulating factor, CXCL8, and CXCR4 (35, 36). One obvious characteristic of ATC is the high percentage of immunosuppressive cytokines (24). By altering the immunological microenvironment, immunotherapy can reinstate the tumor-killing capacity of anti-tumor immune cells (37).

Single-cell RNA sequencing (scRNA-seq) is a novel technology that enables comprehensive analysis of the cell composition and transcriptional phenotype of malignant cells and surrounding immune cells by examining the transcriptome information of individual cells (38). In one study, scholars used scRNA-seq to distinguish tumor cells from normal cells based on differences in copy number (39). They estimated that the average prediction accuracy for identifying tumor cells was 97%. Lu sequenced the single-cell RNA of ATC patients and classified the cell types (40). They divided the cells into eight main types and found that in patients with ATC, the number of endothelial cells decreased significantly, while the number of myeloid cells increased significantly. Furthermore, they also discovered that ATC cells exhibited overexpression of mesenchymal and glial genes. Compared with papillary thyroid carcinoma, the number of M2 macrophages in ATC significantly increased, while the number of M1 macrophages decreased. In ATC tumors, the total depleted cells account for more than 50% of the T cell population. It is also found that TRα1, as a transcription factor, plays a role in inhibiting tumor growth through various signaling pathways. ScRNA-seq revealed that the induction of PAX8 by TRα1 transformed the cell landscape of ATC from one state to another through a transcription program (41). ScRNA-Seq not only deepens our understanding of ATC cell composition and heterogeneity but also offers new insights for personalized therapy and the development of molecular markers.

## 3 Application of immunotherapy in ATC

### 3.1 Immune checkpoint inhibitors

Immune cells have cell surface receptors known as ICIs, which control T lymphocyte activation and effector activities (42). The most well-characterized ICIs are programmed cell death protein 1 (PD-1) and cytotoxic T lymphocyte antigen 4 (CTLA-4). The other ICIs include lymphocyte activation gene 3, T-cell immunoglobulin and immunoreceptor tyrosine-based inhibitory motif domain, T-cell immunoglobulin domain and mucin domain 3 (43). A trial of the PD-1 inhibitor spartalizumab in patients with advanced/metastatic ATC reported an overall response rate of 19%, including a complete response of 7% and a partial response of 12%. PD-L1-positive individuals had a higher response rate (29%) than PD-L1-negative patients (0%) (44). An objective response rate of 16% and a 1-year survival rate of 38% were noted for ATC treated with pembrolizumab or nivolumab (45). However, only one patient in a trial using stereotactic body radiation therapy and the CTLA-4 inhibitor tremelimumab or the PD-L1 inhibitor durvalumab for metastatic ATC lived for more than a year (46).Furthermore, additional research reported the efficacy of pembrolizumab and spartalizumab for patients with ATC, which demonstrated the anti-tumor action of ICIs in ATC management (47, 48).

Before starting immunotherapy, most of the patients in these trials receiving ICIs underwent surgery, radiation, chemotherapy, and even targeted therapy. Six patients with metastatic ATC who had not responded to radiation, chemotherapy, or radioiodine therapy were included in a trial evaluating the combination of lenvatinib and pembrolizumab. The results demonstrated that 66% of the patients had a full response, 16% had stable illness, and 16% had progressing disease. The median progression-free survival for all patients was 16.5 months. Half of the ATC patients were still receiving therapy at the time of data cutoff, with treatment durations of 1–40 months (49). Following surgery, a 67-year-old patient with ATC was treated with the PD-1 inhibitor sintilimab in conjunction with the antiangiogenic drug anlotinib. The patient demonstrated notable tumor shrinkage and an 18.3-month maintained remission (50). After receiving combination therapy consisting of the anti-PD-1 antibody camrelizumab and the multitargeted kinase inhibitor famitinib, a patient with locally advanced unresectable ATC underwent postoperative radiotherapy and complete surgical resection after computed tomography demonstrated a partial lesion response after three cycles. The patient demonstrated a very good quality of life about 24 months after diagnosis (51). Another study reported that three patients with ATC died within six months despite treatment with surgery, radiation, and chemotherapy in addition to pembrolizumab (52). Based on the aforementioned trials, it is believed that the treatment effect might also be connected to the duration of treatment overlap. Hence, more research is needed to fully understand the synergistic mechanism and tolerance of combined treatments. Currently, there are several reports on ongoing ATC studies involving PD-1/PD-L1 inhibitor (NCT05453799, and NCT05119296), or PD-1 combined with other treatment (NCT04171622, NCT04675710, NCT05696548, NCT04238624, NCT05659186, NCT03181100, NCT04400474, and NCT04579757). Please refer to **Tables 1**, **2** for a summary of trials.

**Table 1:** Completed trials of ICIs in ATC.

Title	Population	Treatment	Enrollment	Results	NCT number
PD-1 Blockade in Anaplastic Thyroid Carcinoma	Locally advanced and/or metastatic ATC	Spartalizumab 400 mg IV, once every 4 weeks	42	ORR=19%	NCT02404441
An Evaluation of Clinical Efficacy of Immune Checkpoint Inhibitors for Patients with Anaplastic Thyroid Carcinoma	Locally advanced or metastatic unresectable ATC	Nivolumab,240 or 480 mg IV, every two or four weeks or pembrolizumab, 200 or 400 mg IV, every three or six weeks	13	ORR=16%	Retrospective case
Phase II Trial of Pembrolizumab in Metastatic or Locally Advanced Anaplastic/Undifferentiated Thyroid Cancer	Metastatic or locally advanced anaplastic/undifferentiated thyroid cancer	Pembrolizumab 200 mg IV once every 3 weeks	5	ORR=60%	NCT02688608
Phase 2 Study of Pembrolizumab Combined With Chemoradiation Therapy in Anaplastic Thyroid Cancer	ATC and no prior history of neck radiotherapy	Pembrolizumab, 200 mg intravenously (IV) every 3 weeks, combined with chemoradiotherapy (docetaxel/doxorubicin, 20 mg/m2 each IV weekly plus volumetric modulated arc therapy)	3	ORR=0	NCT03211117
A Pilot Study of Durvalumab (MEDI4736) with Tremelimumab in Combination with Image-Guided Stereotactic Body Radiotherapy in the Treatment of Metastatic Anaplastic Thyroid Cancer	Metastatic anaplastic thyroid cancer	Patients will receive durvalumab and tremelimumab together every 4 weeks (one cycle). SBRT delivered to one metastatic site per standard of care using a standard 9Gy x 3 fractions will be given within 2 weeks after the completion of the first cycle. After 4 cycles, patients will then continue with single agent durvalumab every 4 weeks until disease progression or unacceptable toxicity or a total of 12 months from date of initial treatment.	12	Only 1 patient had stable disease beyond 15 weeks	NCT03122496

ORR, overall response rate; IV, intravenous infusion.

**Table 2:** Ongoing trials of ICIs and combination with other therapies in ATC.

NCT Number	Study Title	Interventions	Enrollment	Phase	Estimated primary completion date
NCT05453799	A Phase II, Multicenter Study of XmAb20717 in Patients With Metastatic Anaplastic Thyroid Cancer With an Exploratory Cohort in Aggressive Hurthle Cell Thyroid Cancer	Vudalimab	54	II	2024-07-15
NCT05119296	Phase II Trial of Pembrolizumab in Metastatic or Locally Advanced Anaplastic/Undifferentiated Thyroid Cancer	Pembrolizumab	20	II	2024-11
NCT04171622	Lenvatinib in Combination With Pembrolizumab for Stage IVB Locally Advanced and Unresectable or Stage IVC Metastatic Anaplastic Thyroid Cancer	Lenvatinib Pembrolizumab	25	II	2025-08-31
NCT04675710	Pembrolizumab in Combination With Dabrafenib and Trametinib as a Neoadjuvant Strategy Prior to Surgery in BRAF-Mutated Anaplastic Thyroid Cancer	Surgery Dabrafenib Pembrolizumab Trametinib	30	II	2024-06-30
NCT05696548	Phase 2 Study of Nivolumab Plus Lenvatinib for Patients With Unresectable Anaplastic Thyroid Cancer (NAVIGATION Study)	Lenvatinib Nivolumab	51	II	2025-07
NCT04238624	A Pilot Study of the Addition of Cemiplimab, an Antibody to PD-1, to the Treatment of Subjects With BRAF-Mutant Anaplastic Thyroid Cancer Who Are No Longer Responding to Dabrafenib and Trametinib	Cemiplimab	15	II	2024-06-20
NCT05659186	A Phase II Study of the Efficacy and Safety of PD-1 Inhibitor and Anlotinib Combined With Multimodal Radiotherapy in the Second-line Treatment of Recurrent or Metastatic Anaplastic Thyroid Cancer	Tislelizumab Anlotinib Radiotherapy	20	II	2024-12-30
NCT03181100	Atezolizumab Combinations With Chemotherapy for Anaplastic and Poorly Differentiated Thyroid Carcinomas	Atezolizumab Bevacizumab Cobimetinib	50	II	2025-07-31
NCT04400474	Exploratory Basket Trial of Cabozantinib Plus Atezolizumab in Advanced and Progressive Neoplasms of the Endocrine System. CABATEN Study	Cabozantinib Atezolizumab	93	II	2023-12
NCT04579757	An Open-Label Phase Ib/II Study of Surufatinib in Combination With Tislelizumab in Subjects With Advanced Solid Tumors	Surufatinib Tislelizumab	135	I/II	2024-04-30

Adverse effects are unavoidable when using ICIs. According to NCT02404441, grade 1 or 2 diarrhea, pruritus, exhaustion, and fever were the most frequent treatment-related adverse effects in patients with advanced/metastatic ATC receiving spartalizumab (44). The most common grade 2–4 adverse events were hypertension, fatigue, weight loss/anorexia, oral mucositis, diarrhea, joint/muscle pain, and hand-foot syndrome in six patients with metastatic ATC who were treated with combined lenvatinib and pembrolizumab after all other forms of surgery, chemotherapy, or radiotherapy had failed. However, the lenvatinib-induced adverse effects necessitated treatment stopping in two patients (49). One patient receiving pembrolizumab treatment developed severe grade 4 colitis (bloody stools, severe dehydration, and hypotension), necessitating extensive fluid resuscitation and systemic steroids (53). Despite the ICI adverse effects reported in several studies, most of them can be alleviated with symptomatic therapy. Therefore, ICIs have emerged as a new tool in ATC treatment.

### 3.2 Adoptive immunotherapy

Adoptive immunotherapy involves injecting immune cells with anti-tumor activity to either directly kill or activate the body’s immune response to kill tumor cells. It includes TILs, T cell receptor engineered T cells (TCR-T), chimeric antigen receptor T cell therapy (CAR-T), and NK therapy. TIL therapy was effective in treating numerous solid tumors, including melanoma, breast, cervical, head and neck, stomach, liver, esophagus, and lung cancers (54). The effect of autologous TILs (LT-145 or LN-145-S1) on patients with ATC and some other cancers is being studied in NCT03449108. As an alternative to TIL therapy, T cells can be isolated from peripheral blood and targeted to tumor cells by transferring synthetic TCR or CAR using gene therapy techniques (55). TCR-T involves transfecting TCR α- and β-chain genes into T cells, which can recognize TSAs. This results in structural alterations in the TCR antigen-binding region of the T cells, enabling them to specifically recognize the corresponding tumor antigens. T lymphocytes expressing specific TCRs can recognize human leukocyte antigen (HLA)–peptide complexes on the surface of tumor cells. Antigenic stimulation signals are transmitted by phosphorylating the intracellular region of the immune receptor tyrosine activation motifs, which activates the T cell immune response (56). Patients with colorectal cancer, synovial sarcoma, and metastatic melanoma demonstrated a notable clinical response to TCR-T. CAR overcomes some of the limitations of TCR. However, the success of CAR-T therapy has been concentrated in hematologic tumors, whereas only partial progression has been observed in solid tumors to date (57, 58). Ongoing studies are evaluating the safety and tolerability of AIC2 CAR-T cells in patients with relapsed/refractory poorly differentiated thyroid cancer and ATC (NCT04420754). Animal studies have demonstrated that adoptive cell therapy based on NKs significantly inhibited metastatic growth in ATC lung metastasis model mice (59). An ongoing study (NCT05194709) is evaluating the efficacy, safety, and pharmacokinetics of anti-5T4 CAR-NK cells in patients with advanced solid tumors, and the NCT03415100 study is evaluating the safety and feasibility of CAR-NK cell therapy targeting NKG2D ligands in patients with metastatic solid tumors.

TIL-treated patients with advanced cutaneous melanoma experienced many adverse events, such as thrombocytopenia, fever, chills, neutropenia, and tachycardia (60). In colorectal cancer, TCR-T causes severe transient inflammatory colitis (61). The most common adverse effects of CAR-T in other tumors are cytokine release syndrome and immune effector cell-associated neurotoxicity syndrome (62). Grade 3 cytokine release syndrome also occurred in two patients in a phase I study of CD33 CAR-NK cells in patients with relapsed or refractory acute myeloid leukemia (63). However, the adverse effects of adoptive immunotherapy in ATC are unclear and require further clarification.

### 3.3 Tumor vaccines

**Figure 1:**
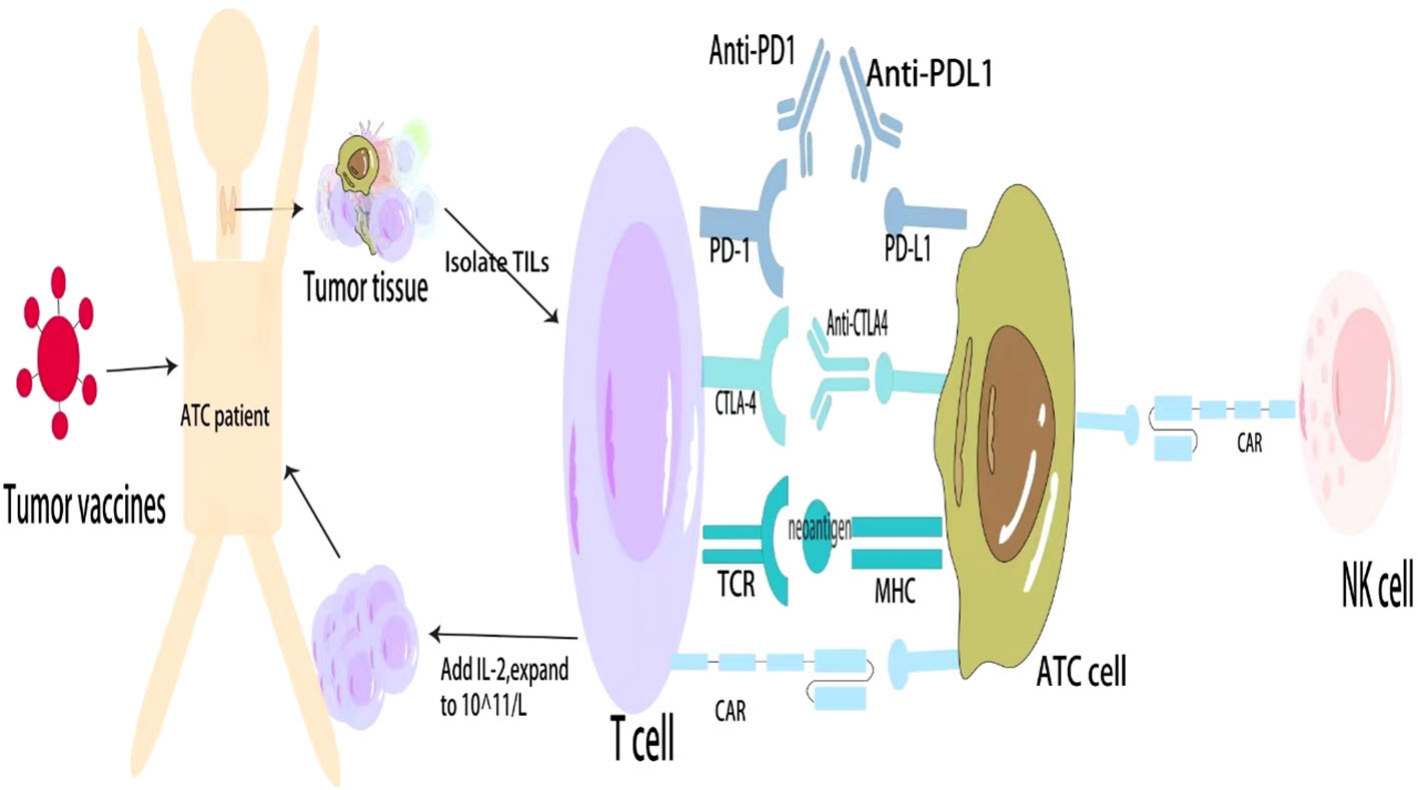
General approaches to immunotherapy for ATC. ATC, Anaplastic thyroid cancer; PD, Programmed cell death protein; CTLA, Cytotoxic T lymphocyte antigen; TCR, T cell receptor; CAR, Chimeric antigen receptor; MHC, Major histocompatibility complex; TILs, Tumor-infiltrating lymphocytes; NKs, Natural killer cells.

Tumor vaccines can stimulate the body to generate active specific immunity against tumor cells or cells or molecules in the TME that promote tumor growth. ATC typically has a high mutation burden, and most of the neoantigens in ATC can be used for tumor vaccines. Thus, tumor vaccines might be more effective in ATC (64). The current ATC vaccines mainly include DC vaccines and oncolytic virus (OV) vaccines. Triiodothyronine can enhance DC ability to stimulate cytotoxic T-cell responses, thereby enhancing anti-tumor responses (65). DC vaccines have been successfully used to treat medullary thyroid carcinoma, and the treatment of thyroid cancer might be more effective than that for other tumors (66). OVs use natural or genetically modified viruses to specifically infect and lyse cancer cells but do not harm normal cells. The anti-cancer activities of OVs are derived from multimodal cancer-killing mechanisms. First, OVs infect and replicate in cancer cells, inducing tumor cell lysis and releasing infectious viral progeny that spreads to the surrounding tumor cells. Second, OV-mediated oncolysis of tumor cells initiates the release of tumor-associated antigens, cellular danger-associated molecular pattern signals, and cytokines that promote the maturation of antigen-presenting cells and activate antigen-specific CD4 and CD8 T cell responses. Third, OVs can specifically infect and destroy tumor vascular endothelial cells and stromal cells and destroy tumor blood vessels by promoting endostatin and angiostatin production (67). Furthermore, OVs induced cell death and tumor regression in cultured ATC cells and mouse models and modulated the ATC microenvironment to shift the M2-type TAMs to the proinflammatory M1 phenotype (68, 69). Although adverse events such as fever, injection site reactions, headache, and vomiting occurred in patients with glioma and melanoma treated with the OV vaccine, they were all tolerable (70). Nevertheless, patients with ATC have not received a tumor vaccine. It is expected that the development of DC And OV vaccines will become a means of treating ATC (**Figure 1**).

## 4 Application limitations and future directions of ATC immunotherapy

Immunotherapy is a promising treatment for patients with ATC, but some clinical management issues persist. ICIs have had tremendous success, but many patients do not respond to them (71). The cell preparation, expansion, and infusion of TILs are complex which can take a long time for patients (72). TCR is limited by human HLA and must match HLA to be effective, whereas CAR-T can recognize extracellular antigens presented independently of HLA (73). However, CAR-T therapy is subject to inadequate infiltration of CAR-T cells, poor proliferation and durability, toxicity control, and an immunosuppressive microenvironment (74). The NK survival rate and cytotoxicity are significantly reduced by the CAR-binding epitope location and its distance from the surface of CAR-NK cells and by NK sensitivity to freeze-thawing (75). DC vaccines are complex and expensive to produce, require ex vivo expansion, maturation, and activation, and have a short half-life *in vivo (* 76). Furthermore, the OV vaccines are subject to drug resistance, and the pharmacokinetics, quality control, and detection methods require further investigation (77, 78).

Immunotherapy is the fourth major therapy for cancer treatment and has achieved good results. However, considering the abovementioned adverse effects and limitations, further development and optimization are required. As the effectiveness of a single treatment is currently restricted, surgery, radiotherapy, chemotherapy, targeted therapy, and immunotherapy can be combined to varying degrees to maximize the advantages of each treatment. Aiming to alleviate symptoms, inhibit tumor cell proliferation, and promote tumor cell apoptosis. Oncolytic herpes simplex virus and BRAF inhibitors enhanced the immune-mediated anti-tumor effect in animal tests, hence improving the survival rate of the ATC mouse model (79). ICIs, targeted therapies, and CAR-T combination therapy demonstrated significant synergistic effects in rectal cancer, non-small cell lung cancer, liver cancer, breast cancer, hematologic neoplasms, and other cancers (80). When the two promising treatments are used in combination, several unknowns remain to be investigated, such as the relative dose, timing, design, and means of overcoming mitigating factors (81). Better preclinical models that more effectively recapitulate the intricate interactions of human immune cells are required to increase the success rate of clinical translation. Given the large presence of TAMs in the ATC microenvironment, targeting TAMs might be a potential ATC treatment. Modifying macrophages with CAR might aid in overcoming immunosuppressive cytokines and upregulate antigen presentation. CAR-M can home in on tumor tissue, have immunity to immunosuppressive TMEs, and lack an immune exhaustion process similar to those of T cells and NKs. Thus, CAR-M might be another potential cell treatment technique (82). CAR-M has been effectively used in animal trials to treat ovarian and breast cancer (83, 84).

## 5 Conclusion

Currently, several studies indicated that immunotherapy can prolong the survival time of patients with ATC, and combining different therapy modalities has greater efficacy than a single-arm treatment. Combination therapy can impede and regulate tumor growth, lower the risk of adverse effects, and exert synergistic anti-tumor effects. Due to the advancements in understanding the mechanisms of immune action, immunotherapy may progress rapidly, which will be excellent news for most patients with cancer.

## Author contributions

JC: Writing – original draft. ZX: Writing – review & editing. HW: Writing – review & editing.

## References

[1] Pacini F Castagna MG Brilli L Pentheroudakis G. Thyroid cancer: ESMO Clinical Practice Guidelines for diagnosis, treatment and follow-up. Ann Oncol Off J Eur Soc Med Oncol. (2012) 23 Suppl 7:vii110–9. doi: 10.1093/annonc/mds230 22997443

[2] Maniakas A Dadu R Busaidy NL Wang JR Ferrarotto R Lu C. Evaluation of overall survival in patients with anaplastic thyroid carcinoma, 2000-2019. JAMA Oncol. (2020) 6:1397–404. doi: 10.1001/jamaoncol.2020.3362 PMC7411939 32761153

[3] Garcia-Alvarez A Hernando J Carmona-Alonso A Capdevila J. What is the status of immunotherapy in thyroid neoplasms? Front Endocrinol. (2022) 13:929091. doi: 10.3389/fendo.2022.929091 PMC9389039 35992118

[4] Ferrari SM Elia G Ragusa F Ruffilli I La Motta C Paparo SR. Novel treatments for anaplastic thyroid carcinoma. Gland Surg. (2020) 9:S28–s42. doi: 10.21037/gs 32055496 PMC6995904

[5] Saini S Tulla K Maker AV Burman KD Prabhakar BS. Therapeutic advances in anaplastic thyroid cancer: a current perspective. Mol Cancer. (2018) 17:154. doi: 10.1186/s12943-018-0903-0 30352606 PMC6198524

[6] Yuan J Guo Y. Targeted therapy for anaplastic thyroid carcinoma: advances and management. Cancers. (2022) 15:179. doi: 10.3390/cancers15010179 36612173 PMC9818071

[7] Pham T Roth S Kong J Guerra G Narasimhan V Pereira L. An update on immunotherapy for solid tumors: A review. Ann Surg Oncol. (2018) 25:3404–12. doi: 10.1245/s10434-018-6658-4 30039324

[8] Wu Z Li S Zhu X. The mechanism of stimulating and mobilizing the immune system enhancing the anti-tumor immunity. Front Immunol. (2021) 12:682435. doi: 10.3389/fimmu.2021.682435 34194437 PMC8237941

[9] Meeusen E Lim E Mathivanan S. Secreted tumor antigens - immune biomarkers for diagnosis and therapy. Proteomics. (2017) 17:1600442. doi: 10.1002/pmic.201600442 28714192

[10] Song Q Zhang CD Wu XH. Therapeutic cancer vaccines: From initial findings to prospects. Immunol Lett. (2018) 196:11–21. doi: 10.1016/j.imlet.2018.01.011 29407608

[11] Yarchoan M Johnson BA 3rd Lutz ER Laheru DA Jaffee EM. Targeting neoantigens to augment antitumour immunity. Nat Rev Cancer. (2017) 17:209–22. doi: 10.1038/nrc.2016.154 PMC5575801 28233802

[12] Zhang Z Lu M Qin Y Gao W Tao L Su W. Neoantigen: A new breakthrough in tumor immunotherapy. Front Immunol. (2021) 12:672356. doi: 10.3389/fimmu.2021.672356 33936118 PMC8085349

[13] Chen F Zou Z Du J Su S Shao J Meng F. Neoantigen identification strategies enable personalized immunotherapy in refractory solid tumors. J Clin Invest. (2019) 129:2056–70. doi: 10.1172/JCI99538 PMC6486339 30835255

[14] Labani-Motlagh A Ashja-Mahdavi M Loskog A. The tumor microenvironment: A milieu hindering and obstructing antitumor immune responses. Front Immunol. (2020) 11:940. doi: 10.3389/fimmu.2020.00940 32499786 PMC7243284

[15] Fidler IJ. The pathogenesis of cancer metastasis: the 'seed and soil' hypothesis revisited. Nat Rev Cancer. (2003) 3:453–8. doi: 10.1038/nrc1098 12778135

[16] Menicali E Guzzetti M Morelli S Moretti S Puxeddu E. Immune landscape of thyroid cancers: new insights. Front Endocrinol. (2020) 11:637826. doi: 10.3389/fendo.2020.637826 PMC8112200 33986723

[17] Caillou B Talbot M Weyemi U Pioche-Durieu C Al Ghuzlan A Bidart JM. Tumor-associated macrophages (TAMs) form an interconnected cellular supportive network in anaplastic thyroid carcinoma. PloS One. (2011) 6:e22567. doi: 10.1371/journal.pone.0022567 21811634 PMC3141071

[18] Jung KY Cho SW Kim YA Kim D Oh BC Park DJ. Cancers with higher density of tumor-associated macrophages were associated with poor survival rates. J Pathol Trans Med. (2015) 49:318–24. doi: 10.4132/jptm.2015.06.01 PMC4508569 26081823

[19] Li Y Jia Y Xu Y Li K. DMF activates NRF2 to inhibit the pro-invasion ability of TAMs in breast cancer. Front Oncol. (2021) 11:706448. doi: 10.3389/fonc.2021.706448 34476214 PMC8406629

[20] Mantovani A Allavena P Marchesi F Garlanda C. Macrophages as tools and targets in cancer therapy. Nat Rev Drug Discov. (2022) 21:799–820. doi: 10.1038/s41573-022-00520-5 35974096 PMC9380983

[21] MacDonald L Jenkins J Purvis G Lee J Franco AT. The thyroid tumor microenvironment: potential targets for therapeutic intervention and prognostication. Hormones Cancer. (2020) 11:205–17. doi: 10.1007/s12672-020-00390-6 PMC7484089 32548798

[22] Chen Y Song Y Du W Gong L Chang H Zou Z. Tumor-associated macrophages: an accomplice in solid tumor progression. J Biomed Sci. (2019) 26:78. doi: 10.1186/s12929-019-0568-z 31629410 PMC6800990

[23] Lv J Liu C Chen FK Feng ZP Jia L Liu PJ. M2−like tumour−associated macrophage−secreted IGF promotes thyroid cancer stemness and metastasis by activating the PI3K/AKT/mTOR pathway. Mol Med Rep. (2021) 24:604. doi: 10.3892/mmr 34184083 PMC8258465

[24] Chakraborty S Carnazza M Jarboe T DeSouza N Li XM Moscatello A. Disruption of cell-cell communication in anaplastic thyroid cancer as an immunotherapeutic opportunity. Adv Exp Med Biol. (2021) 1350:33–66. doi: 10.1007/978-3-030-83282-7_2 34888843

[25] Wang X Zhang Y Zheng J Yao C Lu X. LncRNA UCA1 attenuated the killing effect of cytotoxic CD8 + T cells on anaplastic thyroid carcinoma via miR-148a/PD-L1 pathway. Cancer Immunol Immunother CII. (2021) 70:2235–45. doi: 10.1007/s00262-020-02753-y PMC10992874 33486611

[26] Yin M Di G Bian M. Dysfunction of natural killer cells mediated by PD-1 and Tim-3 pathway in anaplastic thyroid cancer. Int Immunopharmacol. (2018) 64:333–9. doi: 10.1016/j.intimp.2018.09.016 30243069

[27] Visciano C Liotti F Prevete N Cali G Franco R Collina F. Mast cells induce epithelial-to-mesenchymal transition and stem cell features in human thyroid cancer cells through an IL-8-Akt-Slug pathway. Oncogene. (2015) 34:5175–86. doi: 10.1038/onc.2014.441 25619830

[28] Veschi V Verona F Lo Iacono M D'Accardo C Porcelli G Turdo A. Cancer stem cells in thyroid tumors: from the origin to metastasis. Front Endocrinol. (2020) 11:566. doi: 10.3389/fendo.2020.00566 PMC7477072 32982967

[29] Hou Y Wang Q Su L Zhu Y Xiao Y Feng F. Increased tumor-associated mast cells facilitate thyroid cancer progression by inhibiting CD8+ T cell function through galectin-9. Braz J Med Biol Res = Rev Bras pesquisas medicas e biologicas. (2023) 56:e12370. doi: 10.1590/1414-431x2023e12370 PMC10085758 37042867

[30] Mellman I Steinman RM. Dendritic cells: specialized and regulated antigen processing machines. Cell. (2001) 106:255–8. doi: 10.1016/S0092-8674(01)00449-4 11509172

[31] Ugolini C Basolo F Proietti A Vitti P Elisei R Miccoli P. Lymphocyte and immature dendritic cell infiltrates in differentiated, poorly differentiated, and undifferentiated thyroid carcinoma. Thyroid Off J Am Thyroid Assoc. (2007) 17:389–93. doi: 10.1089/thy.2006.0306 17542668

[32] Stassi G Todaro M Zerilli M Ricci-Vitiani L Di Liberto D Patti M. Thyroid cancer resistance to chemotherapeutic drugs via autocrine production of interleukin-4 and interleukin-10. Cancer Res. (2003) 63:6784–90. 14583474

[33] Hwang JH Hwang JH Chung HK Kim DW Hwang ES Suh JM. CXC chemokine receptor 4 expression and function in human anaplastic thyroid cancer cells. J Clin Endocrinol Metab. (2003) 88:408–16. doi: 10.1210/jc.2002-021381 12519884

[34] Pan Z Xu T Bao L Hu X Jin T Chen J. CREB3L1 promotes tumor growth and metastasis of anaplastic thyroid carcinoma by remodeling the tumor microenvironment. Mol Cancer. (2022) 21:190. doi: 10.1186/s12943-022-01658-x 36192735 PMC9531463

[35] Polymeris A Kogia C Ioannidis D Lilis D Drakou M Maounis N. Excessive leukocytosis leading to a diagnosis of aggressive thyroid anaplastic carcinoma: A case report and relevant review. Eur Thyroid J. (2020) 9:162–8. doi: 10.1159/000506767 PMC7265710 32523893

[36] Liu Q Sun W Zhang H. Roles and new insights of macrophages in the tumor microenvironment of thyroid cancer. Front Pharmacol. (2022) 13:875384. doi: 10.3389/fphar.2022.875384 35479325 PMC9035491

[37] Lv B Wang Y Ma D Cheng W Liu J Yong T. Immunotherapy: reshape the tumor immune microenvironment. Front Immunol. (2022) 13:844142. doi: 10.3389/fimmu.2022.844142 35874717 PMC9299092

[38] Wang T Shi J Li L Zhou X Zhang H Zhang X. Single-cell transcriptome analysis reveals inter-tumor heterogeneity in bilateral papillary thyroid carcinoma. Front Immunol. (2022) 13:840811. doi: 10.3389/fimmu.2022.840811 35515000 PMC9065345

[39] Gao R Bai S Henderson YC Lin Y Schalck A Yan Y. Delineating copy number and clonal substructure in human tumors from single-cell transcriptomes. Nat Biotechnol. (2021) 39:599–608. doi: 10.1038/s41587-020-00795-2 33462507 PMC8122019

[40] Lu L Wang JR Henderson YC Bai S Yang J Hu M. Anaplastic transformation in thyroid cancer revealed by single-cell transcriptomics. J Clin Invest. (2023) 133:e169653. doi: 10.1172/JCI169653 37053016 PMC10231997

[41] Hwang E Doolittle WKL Zhu YJ Zhu X Zhao L Yu Y. Thyroid hormone receptor α1: a novel regulator of thyroid cancer cell differentiation. Oncogene. (2023) 42:3075–86. doi: 10.1038/s41388-023-02815-2 PMC12243612 37634007

[42] Li X Shao C Shi Y Han W. Lessons learned from the blockade of immune checkpoints in cancer immunotherapy. J Hematol Oncol. (2018) 11:31. doi: 10.1186/s13045-018-0578-4 29482595 PMC6389077

[43] Luo Y Yang YC Shen CK Ma B Xu WB Wang QF. Immune checkpoint protein expression defines the prognosis of advanced thyroid carcinoma. Front Endocrinol. (2022) 13:859013. doi: 10.3389/fendo.2022.859013 PMC9094437 35574031

[44] Capdevila J Wirth LJ Ernst T Ponce Aix S Lin CC Ramlau R. PD-1 blockade in anaplastic thyroid carcinoma. J Clin Oncol Off J Am Soc Clin Oncol. (2020) 38:2620–7. doi: 10.1200/JCO.19.02727 PMC7476256 32364844

[45] Hatashima A Archambeau B Armbruster H Xu M Shah M Konda B. An evaluation of clinical efficacy of immune checkpoint inhibitors for patients with anaplastic thyroid carcinoma. Thyroid Off J Am Thyroid Assoc. (2022) 32:926–36. doi: 10.1089/thy.2022.0073 35583228

[46] Lee NY Riaz N Wu V Brinkman T Tsai CJ Zhi W. A pilot study of durvalumab (MEDI4736) with tremelimumab in combination with image-guided stereotactic body radiotherapy in the treatment of metastatic anaplastic thyroid cancer. Thyroid Off J Am Thyroid Assoc. (2022) 32:799–806. doi: 10.1089/thy.2022.0050 PMC9293682 35521657

[47] Sukari A Kukreja G Nagasaka M Shukairy MK Yoo G Lin HS. The role of immune checkpoint inhibitors in anaplastic thyroid cancer (Case Series). Oral Oncol. (2020) 109:104744. doi: 10.1016/j.oraloncology.2020.104744 32402656

[48] Shih SR Chen KH Lin KY Yang PC Chen KY Wang CW. Immunotherapy in anaplastic thyroid cancer: Case series. J Formosan Med Assoc = Taiwan yi zhi. (2022) 121:1167–73. doi: 10.1016/j.jfma.2022.01.003 35031200

[49] Dierks C Seufert J Aumann K Ruf J Klein C Kiefer S. Combination of lenvatinib and pembrolizumab is an effective treatment option for anaplastic and poorly differentiated thyroid carcinoma. Thyroid Off J Am Thyroid Assoc. (2021) 31:1076–85. doi: 10.1089/thy.2020.0322 PMC8290324 33509020

[50] Gui L Liu S Zhang Y Shi Y. A remarkable and durable response to sintilimab and anlotinib in the first-line treatment of an anaplastic thyroid carcinoma without targetable genomic alterations: A case report. OncoTargets Ther. (2021) 14:2741–6. doi: 10.2147/OTT.S305196 PMC8068508 33907417

[51] Yang S Ji D Xue F Chen T Wang Y Ji Q. Neoadjuvant famitinib and camrelizumab, a new combined therapy allowing surgical resection of the primary site for anaplastic thyroid carcinoma. Cancer Rep (Hoboken NJ). (2023) 6:e1770. doi: 10.1002/cnr2.1770 PMC9875607 36535914

[52] Chintakuntlawar AV Yin J Foote RL Kasperbauer JL Rivera M Asmus E. A phase 2 study of pembrolizumab combined with chemoradiotherapy as initial treatment for anaplastic thyroid cancer. Thyroid Off J Am Thyroid Assoc. (2019) 29:1615–22. doi: 10.1089/thy.2019.0086 31595822

[53] Spalart V Legius B Segers K Coolen J Maes B Decoster L. Dramatic response to first line single agent pembrolizumab in anaplastic thyroid carcinoma. Case Rep Endocrinol. (2019) 2019:9095753. doi: 10.1155/2019/9095753 31885948 PMC6899289

[54] Hendry S Salgado R Gevaert T Russell PA John T Thapa B. Assessing tumor-infiltrating lymphocytes in solid tumors: A practical review for pathologists and proposal for a standardized method from the international immuno-oncology biomarkers working group: part 2: TILs in melanoma, gastrointestinal tract carcinomas, non-small cell lung carcinoma and mesothelioma, endometrial and ovarian carcinomas, squamous cell carcinoma of the head and neck, genitourinary carcinomas, and primary brain tumors. Adv anatomic Pathol. (2017) 24:311–35. doi: 10.1097/PAP.0000000000000161 PMC5638696 28777143

[55] Bear AS Fraietta JA Narayan VK O'Hara M Haas NB. Adoptive cellular therapy for solid tumors. American Society of Clinical Oncology educational book. American Society of Clinical Oncology. Annual Meeting (2021) 41:57–65. doi: 10.1200/EDBK_321115 34010040

[56] Shafer P Kelly LM Hoyos V. Cancer therapy with TCR-engineered T cells: current strategies, challenges, and prospects. Front Immunol. (2022) 13:835762. doi: 10.3389/fimmu.2022.835762 35309357 PMC8928448

[57] Haslauer T Greil R Zaborsky N Geisberger R. CAR T-cell therapy in hematological Malignancies. Int J Mol Sci. (2021) 22:8996. doi: 10.3390/ijms22168996 34445701 PMC8396650

[58] Martinez M Moon EK. CAR T cells for solid tumors: new strategies for finding, infiltrating, and surviving in the tumor microenvironment. Front Immunol. (2019) 10:128. doi: 10.3389/fimmu.2019.00128 30804938 PMC6370640

[59] Zhu L Li XJ Kalimuthu S Gangadaran P Lee HW Oh JM. Natural killer cell (NK-92MI)-based therapy for pulmonary metastasis of anaplastic thyroid cancer in a nude mouse model. Front Immunol. (2017) 8:816. doi: 10.3389/fimmu.2017.00816 28785259 PMC5519537

[60] Pillai M Jiang Y Lorigan PC Thistlethwaite FC Thomas M Kirillova N. Clinical feasibility and treatment outcomes with nonselected autologous tumor-infiltrating lymphocyte therapy in patients with advanced cutaneous melanoma. Am J Cancer Res. (2022) 12:3967–84. PMC9441996 36119832

[61] Parkhurst MR Yang JC Langan RC Dudley ME Nathan DA Feldman SA. T cells targeting carcinoembryonic antigen can mediate regression of metastatic colorectal cancer but induce severe transient colitis. Mol Ther J Am Soc Gene Ther. (2011) 19:620–6. doi: 10.1038/mt.2010.272 PMC3048186 21157437

[62] Thompson JA Schneider BJ Brahmer J Achufusi A Armand P Berkenstock MK. Management of immunotherapy-related toxicities, version 1.2022, NCCN clinical practice guidelines in oncology. J Natl Compr Cancer Network JNCCN. (2022) 20:387–405. doi: 10.6004/jnccn.2022.0020 35390769

[63] Huang R Wen Q Zhang X. CAR-NK cell therapy for hematological Malignancies: recent updates from ASH 2022. J Hematol Oncol. (2023) 16:35. doi: 10.1186/s13045-023-01435-3 37029381 PMC10082521

[64] Shin E Koo JS. Cell component and function of tumor microenvironment in thyroid cancer. Int J Mol Sci. (2022) 23:12578. doi: 10.3390/ijms232012578 36293435 PMC9604510

[65] Alamino VA Montesinos MM Rabinovich GA Pellizas CG. The thyroid hormone triiodothyronine reinvigorates dendritic cells and potentiates anti-tumor immunity. Oncoimmunology. (2016) 5:e1064579. doi: 10.1080/2162402X.2015.1064579 26942081 PMC4760281

[66] Stift A Sachet M Yagubian R Bittermann C Dubsky P Brostjan C. Dendritic cell vaccination in medullary thyroid carcinoma. Clin Cancer Res an Off J Am Assoc Cancer Res. (2004) 10:2944–53. doi: 10.1158/1078-0432.CCR-03-0698 15131029

[67] Tian Y Xie D Yang L. Engineering strategies to enhance oncolytic viruses in cancer immunotherapy. Signal Transduct Target Ther. (2022) 7:117. doi: 10.1038/s41392-022-00951-x 35387984 PMC8987060

[68] Jiang K Song C Kong L Hu L Lin G Ye T. Recombinant oncolytic Newcastle disease virus displays antitumor activities in anaplastic thyroid cancer cells. BMC Cancer. (2018) 18:746. doi: 10.1186/s12885-018-4522-3 30021550 PMC6052588

[69] Passaro C Borriello F Vastolo V Di Somma S Scamardella E Gigantino V. The oncolytic virus dl922-947 reduces IL-8/CXCL8 and MCP-1/CCL2 expression and impairs angiogenesis and macrophage infiltration in anaplastic thyroid carcinoma. Oncotarget. (2016) 7:1500–15. doi: 10.18632/oncotarget.v7i2 PMC4811476 26625205

[70] Cui C Wang X Lian B Ji Q Zhou L Chi Z. OrienX010, an oncolytic virus, in patients with unresectable stage IIIC-IV melanoma: a phase Ib study. J Immunother Cancer. (2022) 10:e004307. doi: 10.1136/jitc-2021-004307 35383116 PMC8984036

[71] Wang S Xie K Liu T. Cancer immunotherapies: from efficacy to resistance mechanisms - not only checkpoint matters. Front Immunol. (2021) 12:690112. doi: 10.3389/fimmu.2021.690112 34367148 PMC8335396

[72] Wang S Sun J Chen K Ma P Lei Q Xing S. Perspectives of tumor-infiltrating lymphocyte treatment in solid tumors. BMC Med. (2021) 19:140. doi: 10.1186/s12916-021-02006-4 34112147 PMC8194199

[73] Szeto C Lobos CA Nguyen AT Gras S. TCR recognition of peptide-MHC-I: rule makers and breakers. Int J Mol Sci. (2020) 22:68. doi: 10.3390/ijms22010068 33374673 PMC7793522

[74] Jung IY Narayan V McDonald S Rech AJ Bartoszek R Hong G. BLIMP1 and NR4A3 transcription factors reciprocally regulate antitumor CAR T cell stemness and exhaustion. Sci Trans Med. (2022) 14:eabn7336. doi: 10.1126/scitranslmed.abn7336 PMC10257143 36350986

[75] Wang W Jiang J Wu C. CAR-NK for tumor immunotherapy: Clinical transformation and future prospects. Cancer Lett. (2020) 472:175–80. doi: 10.1016/j.canlet.2019.11.033 31790761

[76] Li L Goedegebuure SP Gillanders WE. Preclinical and clinical development of neoantigen vaccines. Ann Oncol Off J Eur Soc Med Oncol. (2017) 28:xii11–xii7. doi: 10.1093/annonc/mdx681 PMC5834106 29253113

[77] Raja J Ludwig JM Gettinger SN Schalper KA Kim HS. Oncolytic virus immunotherapy: future prospects for oncology. J Immunother Cancer. (2018) 6:140. doi: 10.1186/s40425-018-0458-z 30514385 PMC6280382

[78] Shalhout SZ Miller DM Emerick KS Kaufman HL. Therapy with oncolytic viruses: progress and challenges. Nat Rev Clin Oncol. (2023) 20:160–77. doi: 10.1038/s41571-022-00719-w 36631681

[79] Crespo-Rodriguez E Bergerhoff K Bozhanova G Foo S Patin EC Whittock H. Combining BRAF inhibition with oncolytic herpes simplex virus enhances the immune-mediated antitumor therapy of BRAF-mutant thyroid cancer. J Immunother Cancer. (2020) 8:e000698. doi: 10.1136/jitc-2020-000698 32759235 PMC7445339

[80] Li B Jin J Guo D Tao Z Hu X. Immune checkpoint inhibitors combined with targeted therapy: the recent advances and future potentials. Cancers. (2023) 15:2858. doi: 10.3390/cancers15102858 37345194 PMC10216018

[81] Bayat Mokhtari R Homayouni TS Baluch N Morgatskaya E Kumar S Das B. Combination therapy in combating cancer. Oncotarget. (2017) 8:38022–43. doi: 10.18632/oncotarget.v8i23 PMC5514969 28410237

[82] Chen Y Yu Z Tan X Jiang H Xu Z Fang Y. CAR-macrophage: A new immunotherapy candidate against solid tumors. Biomed Pharmacother = Biomedecine pharmacotherapie. (2021) 139:111605. doi: 10.1016/j.biopha.2021.111605 33901872

[83] Klichinsky M Ruella M Shestova O Lu XM Best A Zeeman M. Human chimeric antigen receptor macrophages for cancer immunotherapy. Nat Biotechnol. (2020) 38:947–53. doi: 10.1038/s41587-020-0462-y PMC7883632 32361713

[84] Zhang W Liu L Su H Liu Q Shen J Dai H. Chimeric antigen receptor macrophage therapy for breast tumours mediated by targeting the tumour extracellular matrix. Br J Cancer. (2019) 121:837–45. doi: 10.1038/s41416-019-0578-3 PMC6889154 31570753

